# An Autopsy Case of Rapidly Fulminant Group A Streptococcus Infection in a Previously Healthy 67-Year-Old Woman

**DOI:** 10.7759/cureus.73648

**Published:** 2024-11-13

**Authors:** Daichi Kodama, Akane Onogi, Naoki Watanabe, Takuji Tanaka

**Affiliations:** 1 Department of Diagnostic Pathology (DDP) and Research Center of Diagnostic Pathology (RC-DiP), Gifu Municipal Hospital, Gifu, JPN; 2 Department of Pathology, Takayama Red Cross Hospital, Takayama, JPN

**Keywords:** group a streptococcus, multiple organ failure, necrotizing myositis, polymorphonuclear leukocyte infiltration, soft tissue infection, streptococcal toxic shock syndrome

## Abstract

*Streptococcus pyogenes*, also known as group A *Streptococcus* (GAS), is responsible for various conditions, such as pharyngitis, tonsillitis, necrotizing fasciitis, and streptococcal toxic shock syndrome (STSS). STSS, a rapidly progressing infection involving shock and multi-organ failure, was first reported in Japan in 1992, and since then, the number of cases has been steadily increasing. We herein report an autopsy case of STSS that resulted in sudden death. The patient was a 67-year-old woman who died 100 minutes after walking to the emergency department on her own. This case was characterized by multiple organ failure, septic shock, and a lack of polymorphonuclear leukocyte infiltration in necrotic/infected tissues, which are typical features of STSS. Necrotizing fasciitis of the left lower leg was also identified as a potential cause of STSS in this case. Awareness of this condition is critical, and careful screening and a timely diagnosis are essential to ensure the best possible outcomes for the affected patients. In addition, future vaccination strategies are needed.

## Introduction

Group A *Streptococcus* (GAS; *Streptococcus pyogenes*) is a bacterium that can cause a wide range of diseases, from pharyngitis and tonsillitis to invasive conditions, such as necrotizing fasciitis and extremely rapid and fatal streptococcal toxic shock syndrome (STSS), known as streptococcal toxic shock-like syndrome (TSLS).

STSS, characterized by rapidly spreading GAS infection, shock, and multiple organ failure, is a re-emerging infectious disease first reported in the United States in 1987 [1]. In Japan, the first case was reported in 1992 [2], and the number of infections has recently reached a record high [3]. STSS typically begins with influenza-like symptoms and cutaneous manifestations such as swelling and tenderness, progressing to shock within approximately 24-48 hours [4], and has a high mortality rate of approximately 30% [5]. Owing to its rapid progression and lack of distinctive symptoms, a definitive diagnosis of STSS is challenging, making treatment particularly difficult. Pathologically, STSS is characterized by marked necrosis of soft tissues and the presence of numerous bacteria, with a notable absence of neutrophil response. Cases exhibiting poor neutrophil chemotaxis and infiltration are associated with a poor prognosis [6]. The high pathogenicity of STSS is attributed to factors such as neutrophil necrosis induced by streptolysin O (SLO) and inhibition of neutrophil chemotaxis by interleukin-8 protease (ScpC), leading to disease states characteristic of fulminant infections [7,8].

We report herein an autopsy case of STSS in which the patient died extremely rapidly without an increase in the peripheral white blood cell count. An autopsy revealed marked softening of the spleen, kidneys, heart, and liver. Although numerous bacteria were observed in the lower left leg and other tissues, neutrophil infiltration was absent in the regions.

## Case presentation

Clinical information

A 67-year-old woman with a history of dyslipidemia experienced chills two days before presenting to the hospital (day -2) at noon. On the morning of the following day (day -1), she had a high fever (39.2°C), and diarrhea occurred in the afternoon. In the early morning of the day of presentation (day 0), she developed pain in the left lower extremity and lower abdomen and then presented alone at the Emergency Department on foot. Her level of consciousness was E4V5M6 according to the Glasgow Coma Scale. Her initial vital signs were as follows: heart rate, 91 beats/min; 25 breaths/min; blood pressure, 171/101 mmHg; body temperature, 35.8°C (after taking antipyretics); and oxygen saturation 95% with room air. She was 163 cm tall, weighed 72.4 kg, and had a body mass index of 27.25 kg/m^2^. Tenderness in the left lower leg and edema in both the lower legs were noted. No redness and wounds were observed on the left lower extremity. Laboratory results are presented in Table 1.

**Table 1:** Laboratory data AST: aspartate aminotransferase; ALT: alanine aminotransferase; γ-GT: gamma-glutamyl transferase; LDH: lactate dehydrogenase; CK: creatine kinase; BUN: blood urea nitrogen; CRP: C-reactive protein; Na: sodium; K: potassium

Laboratory Tests		Reference range
Venous Blood Gas Analysis		
pH	7.197	7.35-7.45
Lactate (mmol/L)	8.8	0.5-2.0
Anion Gap (mmol/L)	6.1	10-14
Base Excess (BE, mmol/L)	-12.8	-3.0-3.0
Hematological Analysis		
White Blood Cell Count (/μL)	2.18 × 10³	3.3-8.6 × 10³
Red Blood Cell Count (/μL)	4.68 × 10⁶	386-492 × 10⁶
Hemoglobin (g/dL)	14.3	11.6-14.8
Platelet Count (/μL)	2.3 × 10⁴	15.8-34.8
Biochemical Analysis		
AST (U/L)	214	13-30
ALT (U/L)	101	7-23
γ-GT (U/L)	134	9-32
LDH (IFCC, U/L)	1,283	124-222
Total Bilirubin (mg/dL)	6.3	0.4-1.5
CK	604	41-153
BUN (mg/dL)	29.1	8-20
Creatinine (mg/dL)	2.85	0.46-0.79
CRP (mg/dL)	18.79	0-0.14
Na (mmol/L)	135	138-145
K (mmol/L)	4.7	3.6-4.8
Coagulation Profile		
Prothrombin Time (PT, %)	6	70-200
Activated Partial Thromboplastin Time (seconds)	141.5	24-34
Fibrinogen (mg/dL)	30	170-450
D-dimer (μg/mL)	480	0-1

**Figure 1:**
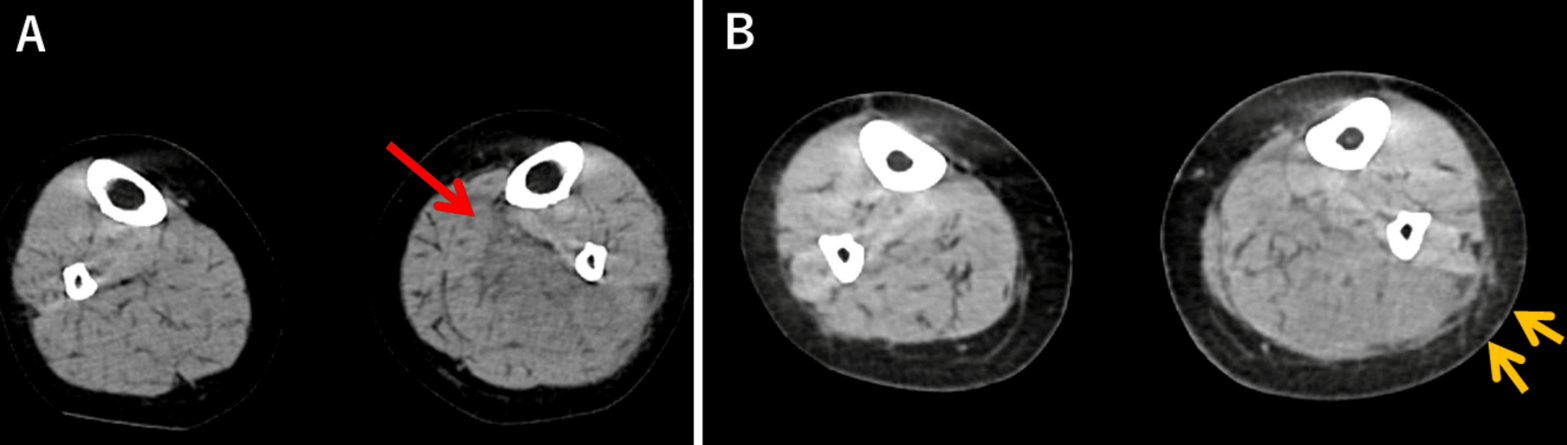
Plain CT of the lower limbs reveals (A) an enlarged left soleus muscle with decreased density (red arrow) and (B) increased density in the perifascial fatty tissue (yellow arrows).

Computed tomography (CT) of the chest, abdomen, pelvis, and lower limbs without contrast showed hypertrophy and decreased density of the left soleus muscle (Figure 1A), along with the increased density of the adipose tissue surrounding the fascia (Figure 1B). Gas formation was not observed in the affected area. The spleen was markedly atrophic. Both lungs demonstrated diffusely increased density with thickening of the interlobular septa and peri-bronchovascular sheaths, suggesting lung edema.

Blood cultures were positive for *S. pyogenes*. A strain analysis performed by Dr. Tadayoshi Ikebe (National Institute of Infectious Diseases, or NIID) revealed that the T serologic typing, T1; the *emm* genotype, *emm1* (100%): the EMM type, EMM1.0 (100%); M1 lineage, M1UK lineage; and the fever toxin gene, positive for *speA*, *speB*, *speC*, *speF*. Although these findings were reported postmortem, the strain in this case of fulminant hemolytic streptococci infection (STR570) was registered with NIID as NIH590.

Based on these findings, the clinical diagnosis was sepsis due to necrotizing fasciitis of the left lower extremity, disseminated intravascular coagulation (DIC), multiple organ dysfunction, and metabolic acidosis. Approximately 70 minutes after admission, her level of consciousness and blood pressure suddenly deteriorated, and hypoxemia developed. Despite immediate tracheal intubation and administration of broad-spectrum antibiotics (meropenem), the patient died approximately 100 minutes after admission.

Autopsy findings

**Figure 2:**
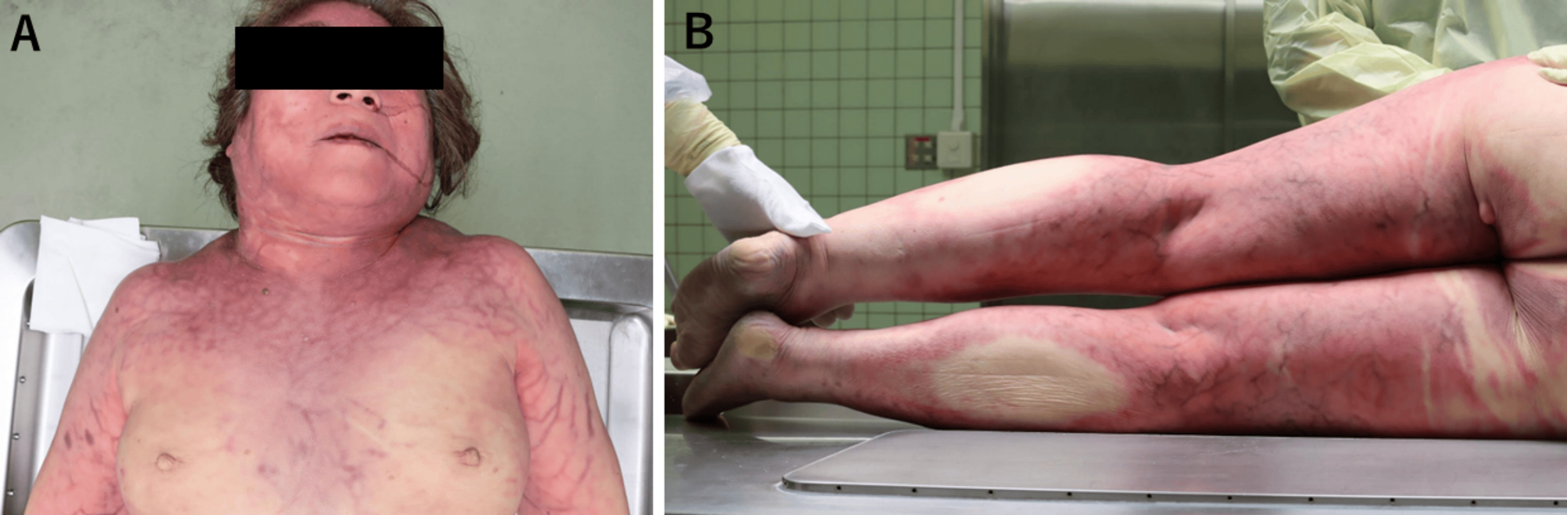
Marked mottling of the skin is observed on the (A) head, neck, upper extremities; and (B) lower extremities.

To clarify the direct cause of death in this case, an autopsy was performed with the consent of the family approximately 25 hours postmortem. The patient was 163.0 cm in height and weighed 72.4 kg. Pronounced mottling of the skin was observed on the head, neck, and upper and lower extremities (Figures 2A-2B).

Organisms

**Figure 3:**
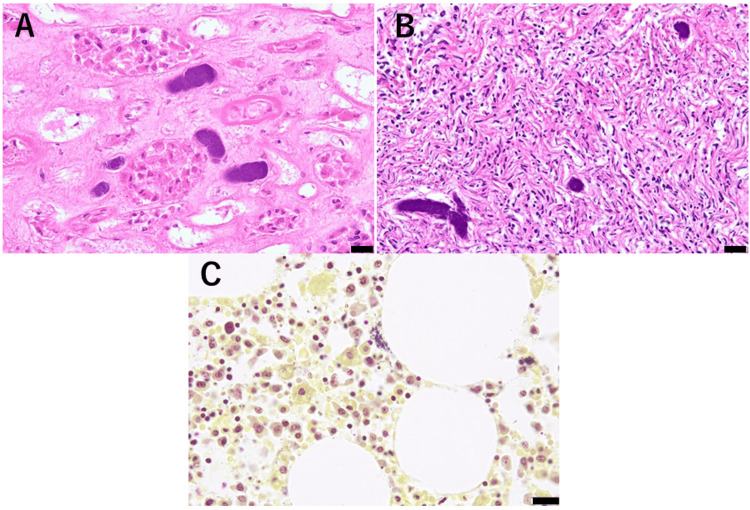
Bacterial aggregates filling the capillaries are observed in the (A) kidney and (B) the ovary (400x magnification). (C) Gram-positive cocci are identified in the bone marrow. (A and B) Hematoxylin and eosin stain, bar: 20 μm. (C) Gram stain, bar: 20 μm.

Bacterial aggregates filling the capillaries were observed in almost all examined tissues (Figures 3A-3B). These Gram-positive cocci (GPC) were also detected within the bone marrow (Figure 3C) and heart (425 g). These findings suggested the presence of bacteremia.

Left Lower Leg

**Figure 4:**
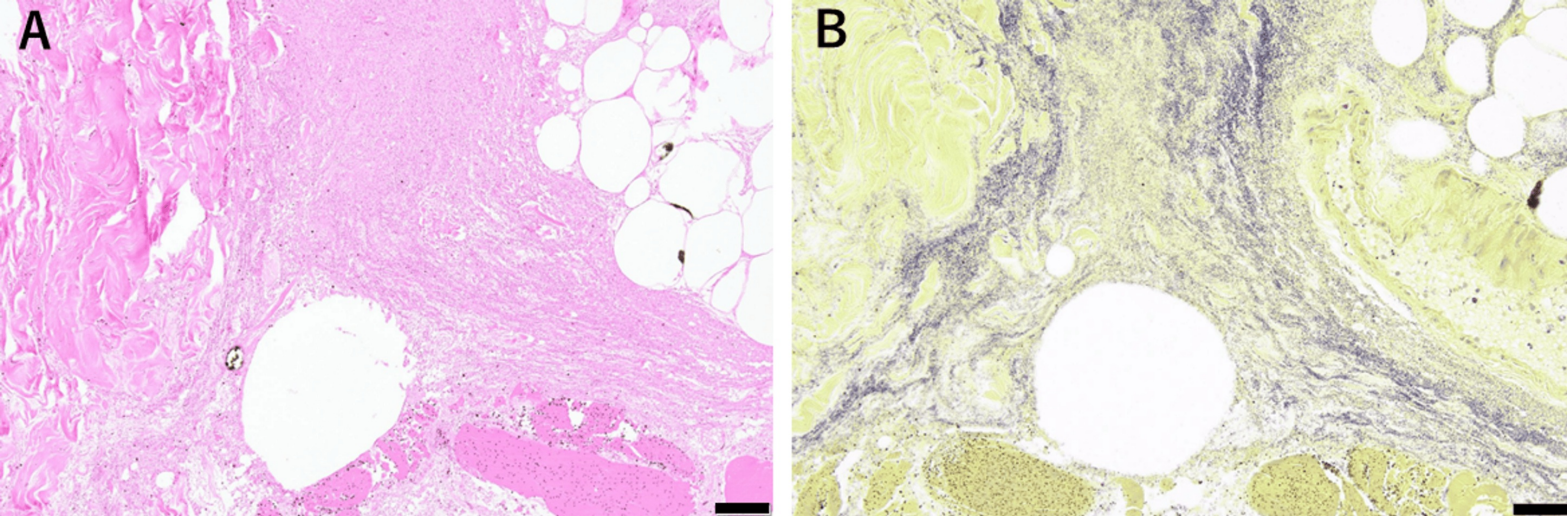
(A) Histopathology of the left lower leg shows necrotizing fasciitis, consisting of necrotic granulation tissue. Note the necrosis without neutrophil infiltration, extending from the deep subcutaneous adipose tissue to the superficial muscle layer (200x magnification). (B) Numerous Gram-positive cocci are observed. (A) Hematoxylin and eosin stain, bar: 50 μm. (B) Gram stain, bar: 50 μm.

Macroscopically, a small blister was observed in the left lower leg. However, no wounds were observed. Microscopically, necrotizing fasciitis composed of scattered GPC and necrotic granulation tissue was identified, extending from the deep subcutaneous adipose tissue to the superficial muscle layers. However, neutrophils’ infiltration was rarely observed (Figures 4A-4B).

Spleen

**Figure 5:**
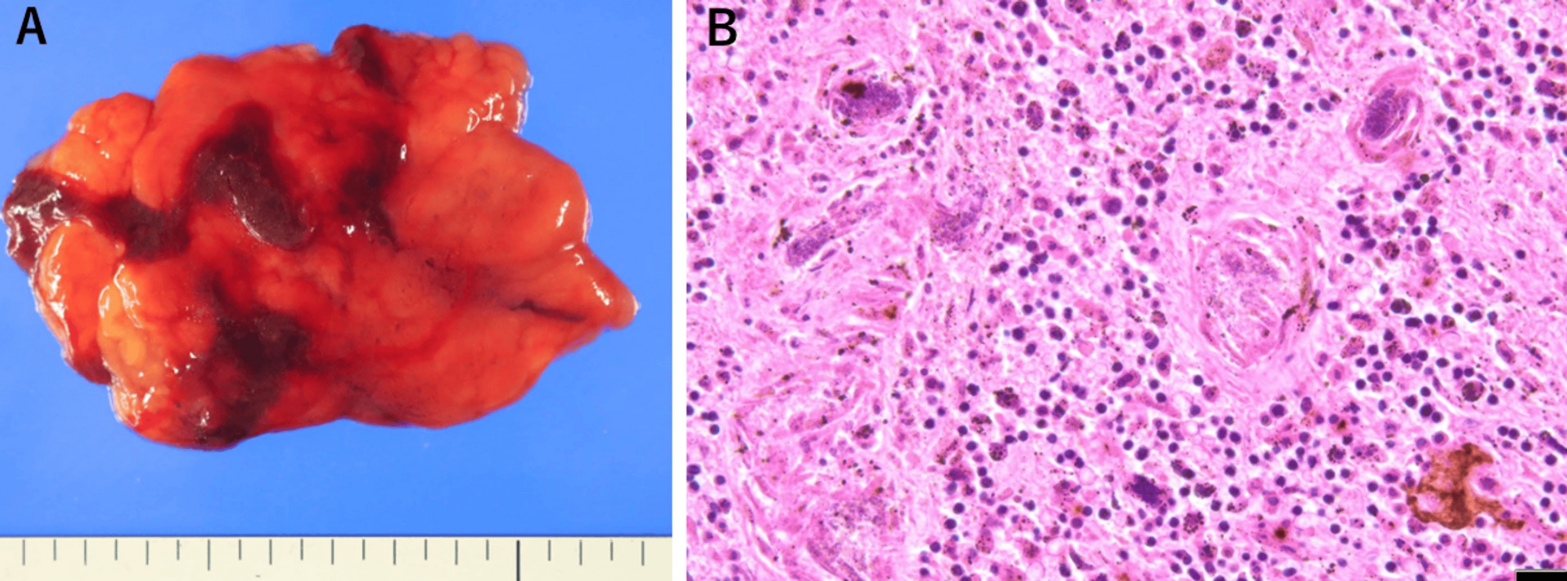
(A) The spleen was markedly softened. (B) Bacterial organisms are observed, but neutrophil infiltration is scarce. (B) Hematoxylin and eosin stain, bar: 20 μm.

The spleen was extremely softened and its structure collapsed when touched (Figure 5A). Therefore, we could not weigh the spleen, and histologically, bacterial organisms were sporadically observed; however, neutrophil infiltration was scarce (Figure 5B).

Others

**Figure 6:**
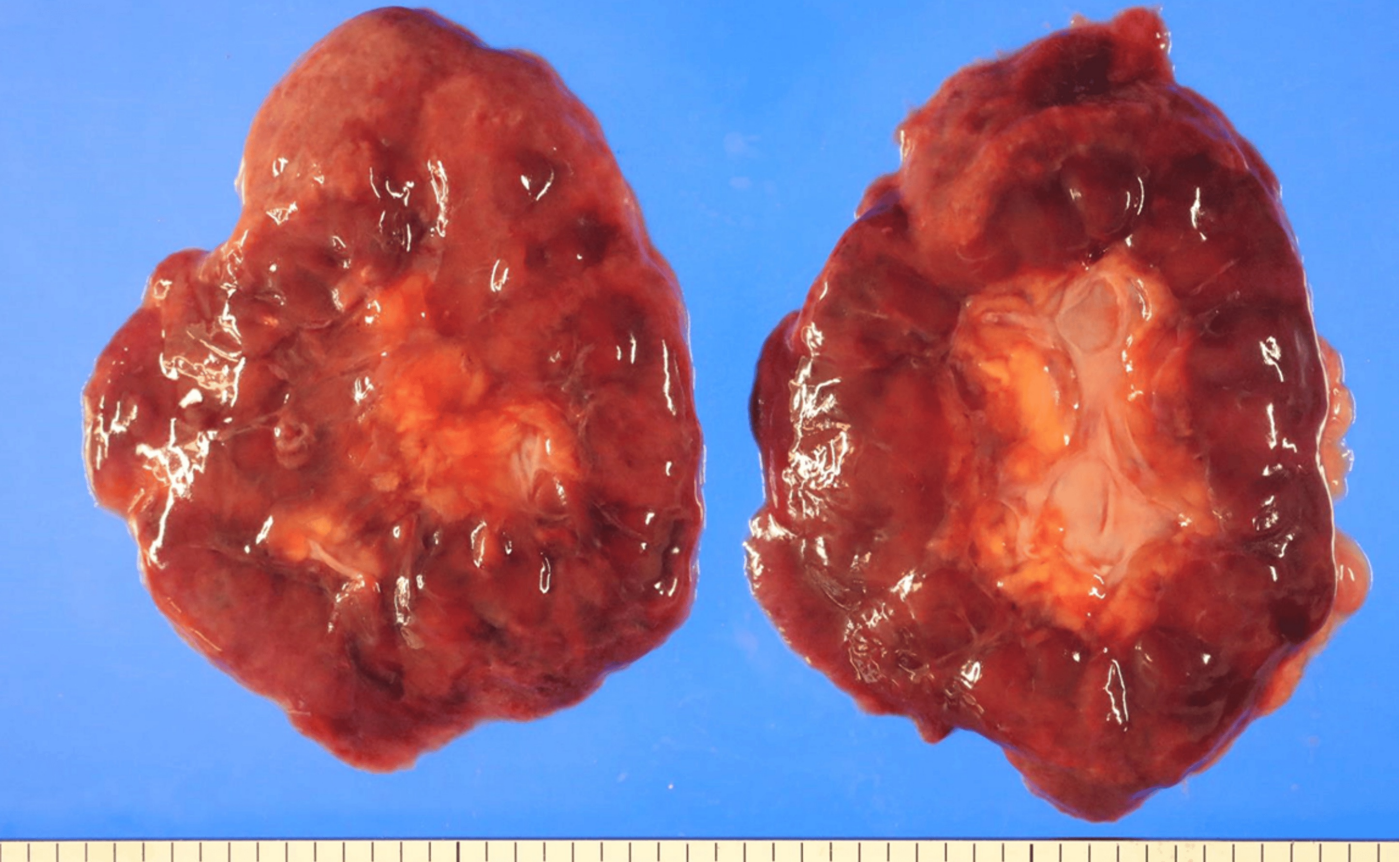
Grossly, the kidneys were extremely softened.

Both the kidneys (140 and 120 g; Figure 6), liver (1,080 g), and heart (425 g) were softened and exhibited a glistening appearance. Histologically, acute tubular necrosis was noted bilaterally in the kidneys. The liver exhibited diffuse hepatocellular necrosis, with a small amount of neutrophil infiltration within the sinusoids. Diffuse alveolar damage (DAD) and pulmonary congestion with edema were observed in the lungs (890 and 880 g, respectively). Bilateral pleural effusion (120 mL and 130 mL) was observed. No pathological findings suggestive of DIC were observed.

Pathological diagnosis

The pathological diagnosis confirmed STSS, characterized primarily by necrotizing fasciitis in the left lower leg and bacteremia. Additionally, sepsis was identified with notable findings that included neutrophil infiltration within the spleen and liver, DAD in the lungs, and acute tubular necrosis in the kidneys. The patient also exhibited mottling of the skin, which is commonly associated with septic shock. Together, these findings illustrate the extensive multi-organ involvement typical of severe STSS cases.

## Discussion

STSS is a re-emerging infectious disease first reported in the United States in 1987 [1]. Between 2003 and 2004, a total of 5,522 cases were reported in 11 European countries (Cyprus, Czech Republic, Denmark, Finland, France, Germany, Greece, Italy, Romania, Sweden, and the United Kingdom) [9]. In Ontario, Canada, the number of cases has been increasing since the initial report in 1990, with 1,745 cases reported between October 2023 and July 2024 [10,11]. In Japan, the first case was reported in 1992 [12], and according to the National Institute of Infectious Diseases, it was reported annually in 2019. Although there was a slight decrease to 600-720 cases per year between 2020 and 2022, by 2023, there were 941 cases (preliminary data), the highest since statistics collection began in 1999. Notably, as of August 27, 2024, there have been 1,364 cases, significantly surpassing the previous highest number of infections [3,12].

In the current case, we noticed necrotizing fasciitis in the left lower leg on autopsy, which was reported as the most common site of tissue infection with GAS [5] with pyogenic exotoxin genes (*speA*, *speB*, *speC*, and *speF*) associated with pathogenicity and mottling of the skin. Furthermore, the patient without underlying diseases developed bacteremia, multiple organ failure, acute tubular necrosis, and DAD. The diagnosis of STSS is extremely difficult as it resembles other systemic inflammatory diseases. Therefore, it is important for clinicians to perform microbiological examinations from the foci of potential infection, even if the initial culture is negative. In the present case, ex-post genotypic analyses showed EMM1.0, which was found to be the predominant emm type followed by 89, 12, and 3 among those identified in more than 70% of STSS isolates in Japan during 2013-2018 [13,14], although the proportion of emm1 decreased from 60.6% during 2010-2013 to 39.3% during 2013-2018 [14]. Continued investigation of the epidemiological changes in GAS that cause STSS can provide useful information for future vaccination strategies and for monitoring the emergence status of antimicrobial-resistant bacteria.

STSS was defined as "an infection with *S. pyogenes* accompanied by sudden onset of shock and organ failure" by the Centers for Disease Control and Prevention (CDC) [15]. STSS typically develops when toxin-producing strains of *S. pyogenes* infect sterile sites such as blood, cerebrospinal fluid, muscle tissue, or pharynx, although the site of bacterial invasion remains unidentified in 35% of cases [5]. The clinical manifestations of STSS are categorized into three stages: the first stage, occurring 24-48 hours prior to the onset of shock, is characterized by influenza-like symptoms (a fever, myalgia, headache, chills), nonspecific gastrointestinal symptoms (nausea, vomiting, diarrhea), and dermatological manifestations (local swelling, tenderness, erythema, blisters); the second stage involving systemic symptoms such as tachycardia, tachypnea, high fever, and the emergence of deep-seated invasive infections such as necrotizing fasciitis; and the third stage marking by sudden and severe shock accompanied by multiple organ failure. Many patients succumb within 24-48 hours after hospital admission [4], with a mortality rate of approximately 30% [5]. Differential diagnoses of STSS include necrotizing fasciitis caused by multiple organisms such as *Enterococcus spp*., viridans group streptococci, *Staphylococcus aureus*, and *Escherichia coli*; *Vibrio vulnificus* infections; STSS; invasive pneumococcal disease; and invasive meningococcal disease [6,16]. Our case must be the third stage. Initial treatment is crucial and includes antimicrobial therapy with penicillin and clindamycin, surgical intervention such as debridement for source control, and supportive care with fluids and vasopressors as necessary [15].

It has been reported that the migration of polymorphonuclear leukocytes defending against infection by hemolytic streptococci is not found in lesions showing the accumulation of the bacteria. This histopathological feature is considered one of the characteristics fulminant form of hemolytic streptococci infection [17]. In the present case, we observed similar findings in several tissues (left lower leg, heart, spleen, liver, and lung). This may suggest that the lack of polymorphonuclear leukocyte infiltration, which is a host-defense factor, in the lesion is particularly important in fulminant form hemolytic streptococci infections. Experimentally, the strains isolated from patients with fulminant form hemolytic streptococci infection reduced the migration ability of neutrophils compared with those isolated from non-fulminant form patients isolates [7]. Even if they migrated, the strains isolated from patients with the fulminant form of hemolytic streptococci infection were reported to deaden most neutrophils [7]. It is well-established that cases exhibiting poor neutrophil chemotaxis and infiltration despite the abundance of bacteria are associated with a poor prognosis [6].

## Conclusions

When patients present with a fever and localized pain, such as lower limb pain, STSS should be considered in the differential diagnosis, even in the absence of visible wounds. It is imperative to assess the characteristic pathological finding of an absent neutrophilic response to bacterial organisms. In addition, during the autopsy of STSS cases, organs may be markedly softened due to septic shock, as observed in this case. Therefore, meticulous and careful handling of organs is crucial for an accurate pathological analysis.
